# Eutrophication governs predator-prey interactions and temperature effects in *Aedes aegypti* populations

**DOI:** 10.1186/s13071-019-3431-x

**Published:** 2019-04-24

**Authors:** Louie Krol, Erin E. Gorsich, Ellard R. Hunting, Danny Govender, Peter M. van Bodegom, Maarten Schrama

**Affiliations:** 1 Institute of Environmental Sciences, Leiden University, Leiden, The Netherlands; 2 Naturalis Biodiversity Centre, Leiden, The Netherlands; 3 The Zeeman Institute for Systems Biology & Infectious Disease Epidemiology Research, University of Warwick, Coventry, UK; 4 School of Life Sciences, University of Warwick, Coventry, UK; 5 School of Biological Sciences, University of Bristol, Bristol, UK; 6 Department of Biology, Woods Hole Oceanographic Institution, Woods Hole, MA USA; 7 Department of Paraclinical Sciences, University of Pretoria, Pretoria, South Africa; 8 Scientific Services Kruger National Park, Skukuza, South Africa

**Keywords:** Ecological drivers, Vector-borne, Anthropogenic pressures, Interaction effects, Temperature, Biodiversity decline

## Abstract

**Background:**

Mosquito population dynamics are driven by large-scale (e.g. climatological) and small-scale (e.g. ecological) factors. While these factors are known to independently influence mosquito populations, it remains uncertain how drivers that simultaneously operate under natural conditions interact to influence mosquito populations. We, therefore, developed a well-controlled outdoor experiment to assess the interactive effects of two ecological drivers, predation and nutrient availability, on mosquito life history traits under multiple temperature regimes.

**Methods:**

We conducted a temperature-controlled mesocosm experiment in Kruger National Park, South Africa, with the yellow fever mosquito, *Aedes aegypti.* We investigated how larval survival, emergence and development rates were impacted by the presence of a locally-common invertebrate predator (backswimmers *Anisops varia* Fieber (Notonectidae: Hemiptera), nutrient availability (oligotrophic *vs* eutrophic, reflecting field conditions), water temperature, and interactions between each driver.

**Results:**

We observed that the effects of predation and temperature both depended on eutrophication. Predation caused lower adult emergence in oligotrophic conditions but higher emergence under eutrophic conditions. Higher temperatures caused faster larval development rates in eutrophic but not oligotrophic conditions.

**Conclusions:**

Our study shows that ecological bottom-up and top-down drivers strongly and interactively govern mosquito life history traits for *Ae. aegypti* populations. Specifically, we show that eutrophication can inversely affect predator–prey interactions and mediate the effect of temperature on mosquito survival and development rates. Hence, our results suggest that nutrient pollution can overrule biological constraints on natural mosquito populations and highlights the importance of studying multiple factors.

**Electronic supplementary material:**

The online version of this article (10.1186/s13071-019-3431-x) contains supplementary material, which is available to authorized users.

## Background

Mosquitoes are important disease vectors globally, and as such, mosquito population dynamics receive substantial significant scientific attention [1–4]. Although it is widely acknowledged that mosquito population dynamics are driven by large-scale climatological conditions (temperature, precipitation) [5, 6], there is a growing awareness that mosquitoes inhabit complex ecosystems and are, therefore, exposed to a myriad of biotic factors (bottom-up, e.g. resource availability and top-down, e.g. predation) that also influence the success of mosquito-populations [7–10]. Accurate information on mosquito population dynamics has been shown to improve predictions of the timing, likelihood, or location of mosquito borne-disease outbreaks [2, 3, 11]. It is, therefore, crucial to understand both the local ecological context and large-scale climatological conditions in which disease transmitting mosquito vector populations thrive.

Previous studies have demonstrated that eutrophication and predation can present important bottom-up and top-down controls of local mosquito populations [8, 10, 12]. Eutrophication and the resulting increase in food availability for mosquito larvae increases their developmental rates, thus promoting higher numbers of adults emerging from temporary ponds [12, 13]. In contrast, the presence of predators can diminish population sizes of their prey [10, 12, 14]. For instance, a number of species belonging to Hemiptera prey on *Aedes aegypti* larvae [15–17]; their introduction in car tires and other artificial *Ae. aegypti* breeding habitats can reduce adult mosquito abundances by 95% after one year [18]. Although the separate effects of eutrophication and predation on mosquito populations is well known, mounting evidence from alternative systems highlights the importance of studying their combined consequences of mosquito populations. Interactions between multiple ecological drivers can modify not only the strength but also the direction of outcomes at the population or community level [19, 20] and ecological change is often associated with shifts in more than one potential driver. Taken together, this implies that it is essential to study multiple biotic and abiotic drivers of mosquito populations simultaneously under the highest possible degree of natural realism.

The mosquito *Ae. aegypti* is one of the most widespread vector species worldwide [6] and is able to transmit a wide range of viral pathogens such as chikungunya, yellow fever, Zika, dengue and Rift Valley fever (e.g. [21]). While container habitats in urbanized areas (flower vases, buckets with stored tap water, jerry cans, car tires) are generally considered to be the predominant hatching sites of *Ae. aegypti* [22], source population in natural and rural sites adjacent to urbanized areas present another class of environments where they can be found in high abundances [23, 24]. Habitats include mud pots, rock pools, large dead leaves, bromeliads, and tree holes, which sometimes contain large amounts of leaf litter and varying sources of pollution [23, 25–28]. These habitat classes vary not only in their inherent food availability, but also in abundance of predators [29, 30]. Pan-globally, natural predators of *Ae. aegypti* (e.g. various *Anisops* spp.) occur in a wide variety of temporary habitats that exhibit varying levels of nutrients [31, 32]. As such, natural populations of *Ae. aegypti* are simultaneously governed by both nutrients and predators. Here, we use *Ae. aegypti* in a novel, well-controlled outdoor mesocosm setup to assess the interactive effects of eutrophication and predation on mosquito populations under four different temperature regimes.

## Methods

### Study design

**Fig. 1:**
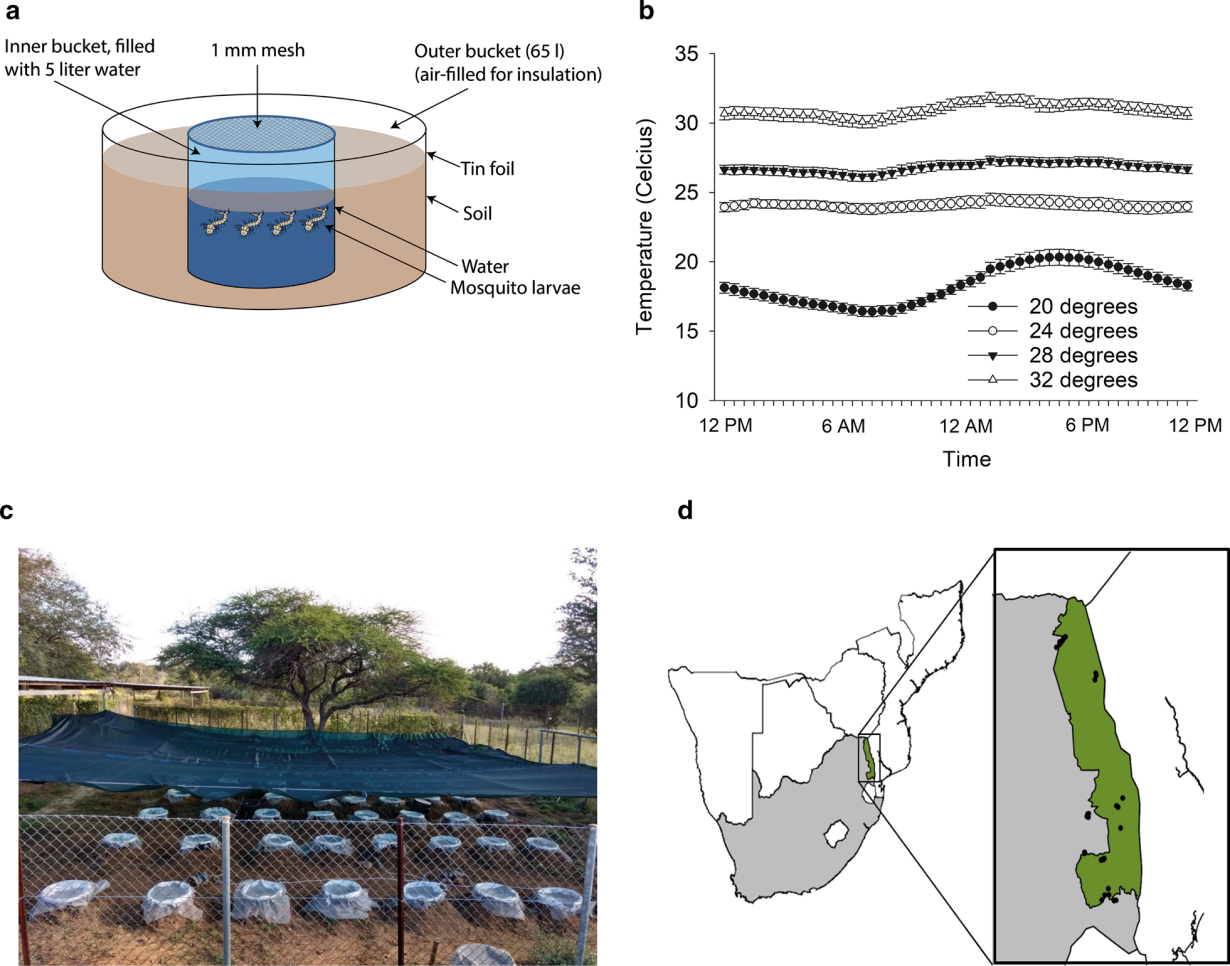
Setup of the mesocosm experiment in Skukuza, Kruger National Park. **a** Schematic drawing of a mesocosm, courtesy of Erik-Jan Bosch. **b** Mean temperatures in each of the temperature treatments ± standard error (SE), as measured with i-buttons in the different mesocosms during the entire period of the mesocosm experiment. Except for the lowest temperature treatment, all treatments contained aquarium heaters. **c** Overview of the setup. **d** Locations in and around Kruger National Park (indicated with red dots) where concentrations of inorganic phosphorus were measured to determine the median eutrophication status for the experiment

The experiment was conducted between 5th and 19th May 2017 in 48 mesocosms at an outside, fenced facility at Skukuza, Kruger National Park, South Africa. Each of the mesocosms consisted of a 48-litre polyethylene tub, which was dug 20 cm into the soil and wrapped with tin foil to prevent heating. A 12-litre bucket was fitted into it, which was filled with 5 litres of rain water (Fig. 1a). This design was developed to prevent the absorption of external heat and enables a buffered micro-climate and precise temperature control in the inside buckets (Fig. 1b). The entire experimental area was covered with 80% shade cloth (Fig. 1c). Prior to setting up the treatments, all 12-litre inside buckets were rinsed thrice, using 1 litre of dechlorinated tap water. Five litres of dechlorinated tap water were added to the 12-litre internal buckets. All mesocosms were covered with mesh (Ø 1 mm) to prevent animal escapes or introductions.

### Rearing of mosquito larvae

To cultivate sufficient numbers of larvae, roughly 4000 *Ae. aegypti* eggs were distributed over 5 small white plastic containers (15 × 10 × 12 cm). One day after hatching, larvae were fed once, using a modified version of the protocol by Zheng et al. [33]. In short, 250 mg of Mnanti beer powder was mixed with 0.7 l of chloride-free water. Each small container included approximately 800 eggs and received 125 ml of this mixture. After six days at 20 °C and a natural day-night light regime, larvae were transferred to the mesocosms.

### Treatments

A mix of first and second instar mosquito larvae (25 individuals) were exposed to a full factorial design including temperature (4 levels: ambient temperature and three treatments at progressive increments), eutrophication (2 levels, oligotrophic/eutrophic) and predation (2 levels, present/absent). This amounted to 16 different treatments in triplicate (*n* = 48). Within each of the three blocks all 16 treatments were randomized (Additional file 1: Figure S1).

#### Temperature

Four temperature regimes were created using aquarium heaters (50W, Aquadistri UK Ltd., Great Gransden, UK), which were placed at an angle of 30 degrees. The treatment with no heaters reflected the ambient temperature. Heaters were set to respectively 24, 28 and 32 °C six days prior to adding the mosquito larvae and were slightly adjusted in the days after to ensure that temperatures within each mesocosm reflected the desired treatment value. Temperature was monitored throughout the study period using i-buttons (Maxim Corp., San Jose, USA). The average mesocosm temperature in this experiment was calculated by taking the mean across days and replicate mesocosms (Fig. 1b). This resulted in mean temperatures of 18.4, 24.1, 26.8 and 30.9 °C.

#### Eutrophication

To set up eutrophication levels that mimicked those in water bodies in and around Kruger National Park, inorganic phosphorus was measured in 36 locations between 18th March and 7th May 2017 (Fig. 1d). The median levels of inorganic phosphorus in eutrophic natural water bodies was 1.025 mg l^−1^, as determined using a photospectrometer (Spectroquant Nova 60; Merck, Darmstadt, Germany) using a phosphate cell test for 0.05–5.0 mg l^−1^ PO_4_^2−^. To mimic these levels, we used a slurry of tap water and impala faeces (*Aepyceros melampus* Petersi). A calibration curve was constructed to calculate the amount of faeces needed to mimic the median concentration in natural situations (Additional file 1: Figure S2). The eutrophication treatment mesocosms were spiked with the impala faeces-based slurry one day prior to the addition of mosquito larvae (t = 0 days). To determine effectiveness of the treatments, phosphate (PO_4_) and nitrate (NO_3_) concentrations were measured in the mesocosms (t = 12 days) using a similar procedure as described above. In mesocosms not receiving the eutrophication treatment, the so-called oligotrophic treatment, no food was added to mimic rainwater fed breeding containers with realistic nutrient availability [12].

#### Predation

Predation pressure was investigated by adding one adult *Anisops varia* Fieber (Hemiptera: Notonectidae) to half of the mesocosms one day after the mosquito larvae had been added (t = 2). This species is a common freshwater invertebrate carnivore species in South Africa in a variety of water types, including container habitats [32], which, like other Notonectidae species, has a feeding preference for mosquito larvae and other small invertebrates in the water column [34]. Individuals (*n* = 24) were collected from a nearby temporary pond. We focused on this species because other well-known predators of mosquito lar vae such as Belastomitidae (*Diplonychus* sp.) or Culicidae (*Toxorhynchites* sp.) were not present in the area. In five mesocosms, predators died during the experiment and were replaced the same day. During the experiment, three *A. varia* individuals were observed on top of the mesh covering the mesocosms (Fig. 1a) in an apparent attempt to colonize the mesocosms, thus highlighting their ability to colonize such temporal ponds.

#### Mosquito life history parameters

Emergence of the first *Ae. aegypti* male was observed 4 days after the experiment started, after which we recorded the number of emerged adult mosquitoes from all mesocosms on a daily basis. The daily number of emerging adults was assessed using a manual aspirator. Newly emerged mosquitoes were sexed and counted. The experiment was terminated at t = 19 days because the majority of adults generally emerge at temperatures above 20 °C [35]. However, some mesocosms still contained a fraction of the pupae and larvae, which we expected to be an effect of the experimental conditions. At t = 19 days, water was filtered using a plankton net (Ø 0.5 mm) to count the remaining *Ae. aegypti* larvae and pupae in the mesocosms. We present the cumulative number of emerged male and female adult mosquitoes at t = 14 days and average larval development rate at day 14. The average larval development rate was calculated as 1/(average number of days until between egg and emergence of a given mesocosm) and daily survival was calculated as 1/(number of emerged adults/number of counted larvae at t = 14 days of a given mesocosm). Because some mesocosms had no adults emerging or larvae/pupae remaining at the termination of the experiment, we were also interested in how the treatments affected these parameters.

### Data analysis

First, we explored the effectiveness of the temperature, eutrophication and predation treatments on abiotic factors in the mesocosms. To confirm that the temperature treatments were effective, we tested the temperature differences between the four temperature regimes with a one-way ANOVA and a post hoc Tukey test. The dependent variable was the average temperature value for each mesocosm across the study. Differences in abiotic factors (phosphate concentration (PO4), nitrate concentration (NO_3_), pH, total dissolved salts and electrical conductivity (EC)) were tested with a general linear model with categorical variables representing each mesocosm’s temperature treatment (4 levels, see above), predation treatment (2 levels: present/absent), eutrophication treatment (2 levels: oligotrophic/eutrophic) and their 2-way interactions as a fixed factor.

Secondly, we explored the effects of temperature, eutrophication and predators and their interactions on seven *Aedes*-related outcomes at t = 19. These were: (i) cumulative number of emerged male adult mosquitoes at t = 19; (ii) cumulative number of emerged female adult mosquitoes at t = 19; (iii) total cumulative number of emerged adults; (iv) whether or not any adults emerged before t = 19 (yes/no); (v) development rate (1/days between egg and emergence); (vi) number of remaining larvae and pupae at t = 19; and (vii) whether or not larvae or pupae remained at t = 19 (yes/no).

Effects of eutrophication, temperature and predation on each outcome were tested with linear models and post hoc Tukey HSD tests and their 2-way interactions as fixed factors. Block was added as a random variable. Effects of the experimental treatments on the odds that larvae survived and adults emerged were tested in two separate analyses using a logistic regression model with the odds of emergence as binomial response variables and the experimental treatments (eutrophication, predation and temperature) as fixed factors. Block was again included as a random variable.

## Results

### Experimental conditions

**Fig. 2:**
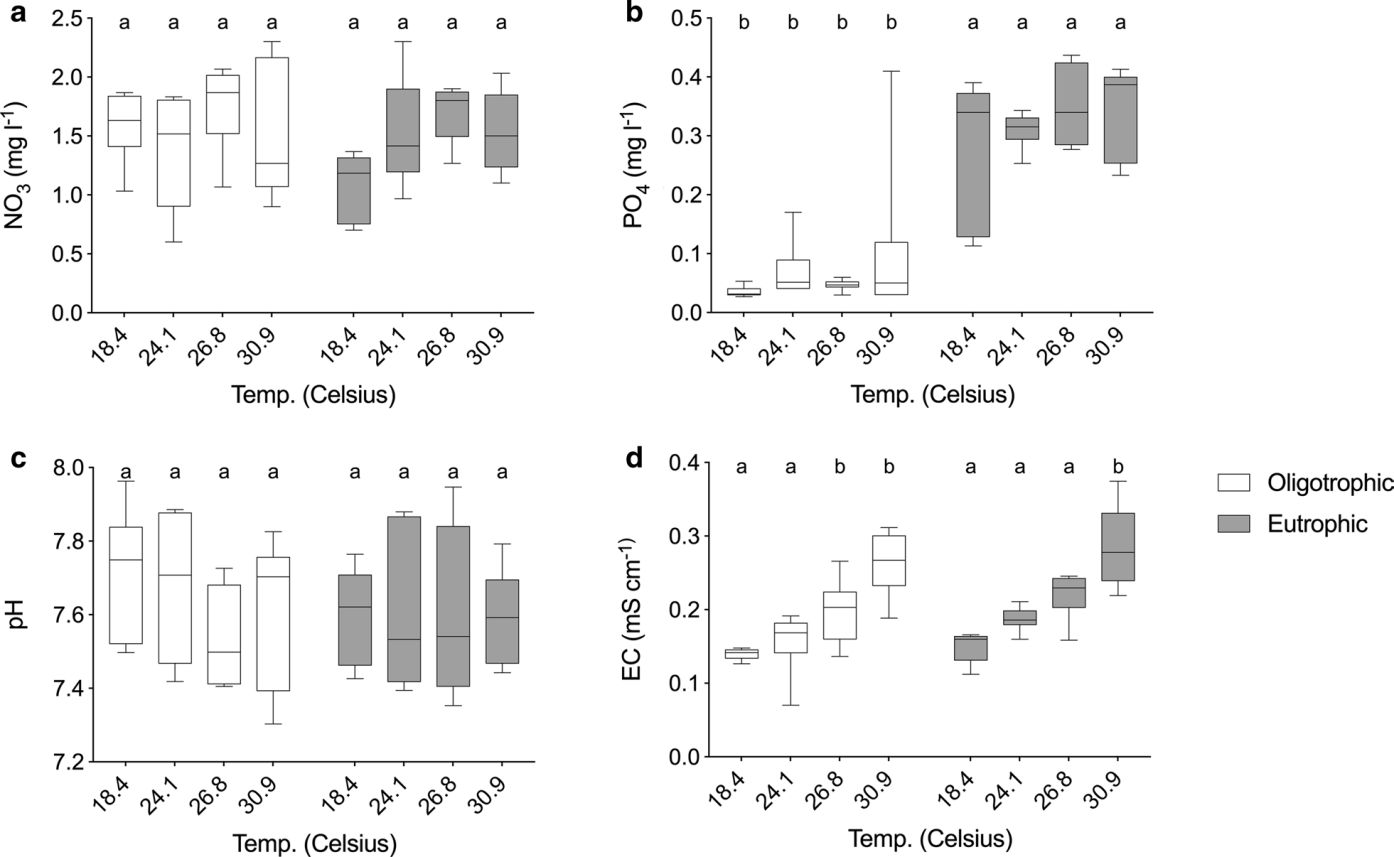
Overview of eutrophication variables (**a**, **b**) (NO_3_, PO_4_) and abiotic variables (**c**, **d**): pH; tds, total dissolved salts; EC (mV), electro conductivity in mS per cm. All data shown as the mean ± standard error (SE). Treatments with and without predators were merged in this table. Different letters indicate significant differences between treatments at α = 0.05

Analyses of abiotic conditions support the validity of our outdoor experimental design. Mesocosms that received the eutrophication treatment contained higher concentrations of phosphorus (PO_4_), but there was no difference in nitrate (NO_3_) concentrations (Fig. 2). Moreover, there was no significant effect of eutrophication on EC, total dissolved salt or pH (Fig. 2). We found no effect of predation on any of the abiotic factors. Temperature was significantly different among temperature treatments (Fig. 1b) and significant effects were observed for some abiotic factors. The two highest temperate treatments had a higher EC and total dissolved salts, but we found no effect of temperature on nutrient concentrations (PO_4_, NO_3_) and pH (Fig. 2). Moreover, there were no significant interaction effects between treatments for each of the abiotic factors.

In 12 mesocosms, no emergence of mosquitoes occurred, 11 of which belonged to the oligotrophic treatment. These 12 mesocosms were excluded from the calculations on development rate but were included in the analyses of adult emergence and the number of larvae remaining.

### Effects on adult emergence

**Fig. 3:**
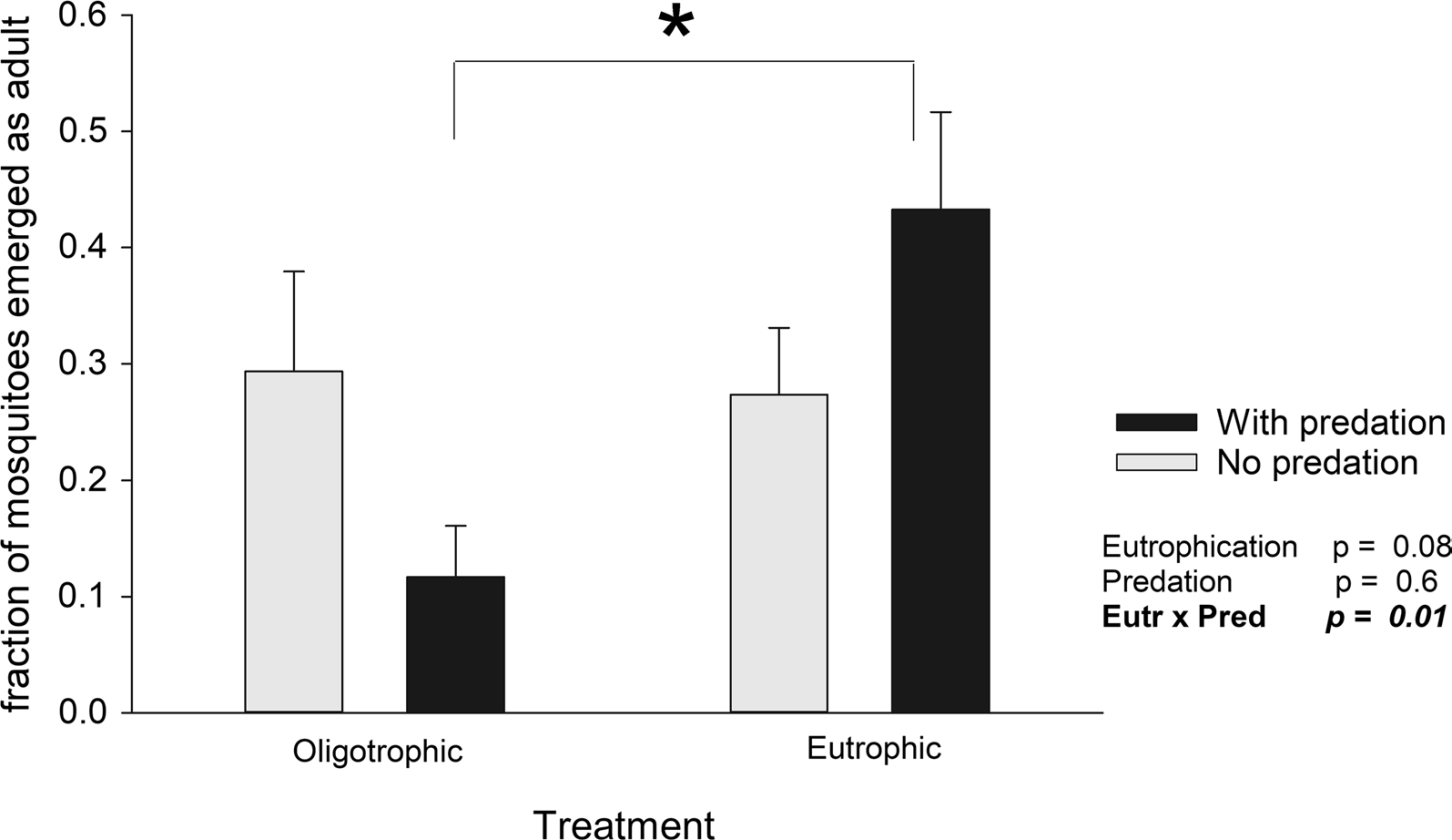
Effect of eutrophication and predation as interacting pressures on emergence of adult *Ae. aegypti*. Bars indicate average cumulative emergence across all temperature regimes ± standard error (SE). Although there was a significant effect of temperature influencing the number of adults emerged, there were no significant interactions between temperature and eutrophication/predation, such that the pattern shown in the figure is representative across temperature treatments. Fractions are shown for illustration purposes only; statistics were done on the cumulative number of emerged adults. Star indicates a significant difference at α = 0.05

The number of emerged adults ranged from 0 to 25, resulting in average emergence of 6.9 individuals (± SD 6.5). The cumulative number of adult mosquitoes which had emerged from a mesocosm at t = 19 was affected by the interaction between eutrophication and predation (*F*_(1,36)_ = 6.0, *P* = 0.02; Fig. 3). Predation in oligotrophic conditions resulted in a 60% *decrease* in adult emergence, whereas predation in eutrophic conditions resulted in a 30% *increase* of adult emergence (Fig. 3). The number of emerging adult mosquitoes was also affected by temperature (*F*_(3,36)_ = 3.8, *P *= 0.02; Additional file 1: Table S1), with the highest number of mosquitoes emerged at intermediate temperatures (Additional file 1: Figure S3). Of the 12 mesocosms where no adult emergence was observed, six were of the lowest temperature treatment, four were exposed to the highest temperature and one belonged to each of the intermediate temperature treatments (24 and 28 °C), suggesting a negative effect of high and low temperatures on adult emergence.

**Fig. 4:**
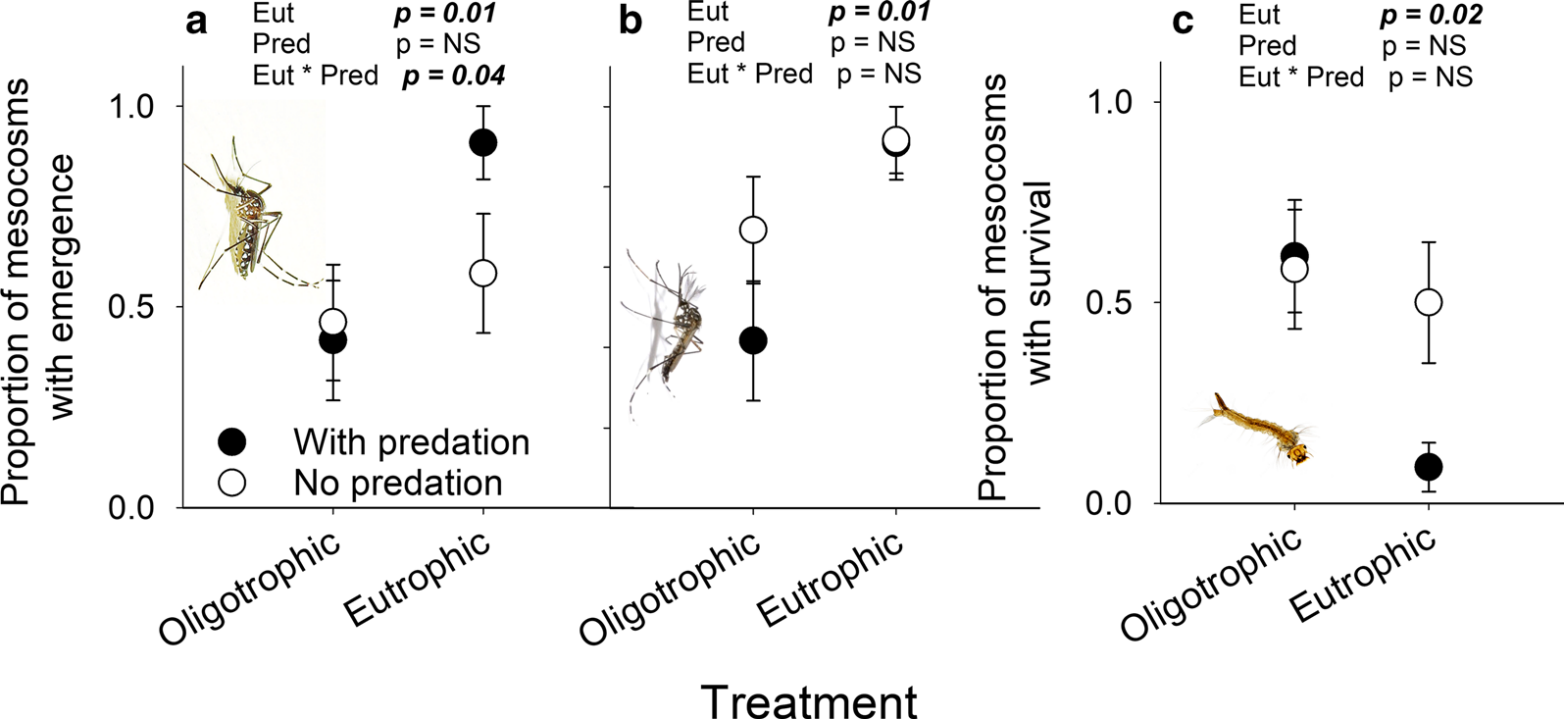
Effect of eutrophication and predation treatments on the odds of emergence of females (**a**), emergence of males (**b**) and larval survival (**c**). Statistics shown above each of the panels depict results from a logistic regression model. Each symbol represents the average across all temperature regimes. NS indicates *P* > 0.05

We found a positive effect of eutrophication (Wald Stat = 6.9, *P* = 0.010) and a significant interaction effect (Wald Stat = 4.9, *P* = 0.038) between predation and eutrophication on the odds that females emerged and a positive effect of temperature on the odds that females emerged (Wald Stat = 4.8, *P* = 0.028; Fig. 4a). The odds that males emerged, which happens in general 1–2 days before females emerge, was significantly and positively affected by eutrophication (Wald Stat = 6.8, *P* = 0.009; Fig. 4b) and not by temperature or predation (Fig. 4b).

### Effects on development rate

**Fig. 5:**
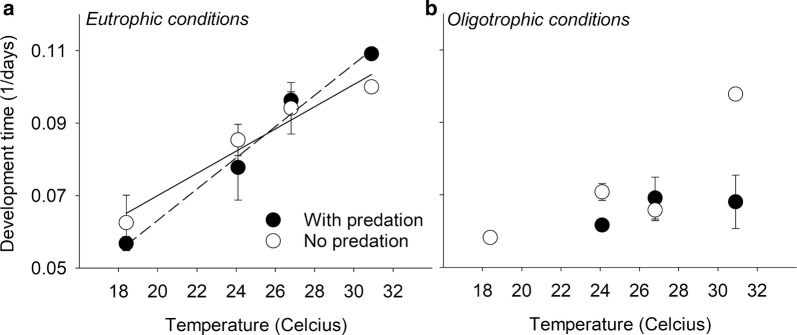
Effect of eutrophication, temperature and predation on development rate of *Aedes aegypti*. **a** Results for eutrophic conditions. **b** Results for oligotrophic conditions. Fits in **a** are for illustrative purposes only

The time to emergence ranged between 9 and 19 days, with an average 13.9 days (± SD 3.16). We found no significant effect of predation on development rate. However, there was a significant effect of eutrophication (*F*_(1,21)_ = 8.7, *P* = 0.007), an effect of temperature (*F*_(3,21)_ = 5.8, *P* = 0.005), and a significant interaction effect between eutrophication and temperature (*F*_(3,21)_ = 7.7, *P *= 0.004) on development rate. The association between development rates and temperature was stronger under eutrophic conditions than under oligotrophic conditions (Fig. 5a, b).

### Effects on surviving larvae

On average, 12.9% (± SE 3.3%) of the larvae remained in the mesocosms (i.e. survived as larvae and did not emerge as adults) after the experiment was terminated at t = 19. The odds that larvae remained in the mesocosms until the end of the experiment was lower under eutrophic conditions (Wald Stat = 5.6, *P* = 0.018) and higher temperatures (Wald Stat = 5.2, *P* = 0.023), but was not significantly affected by predation (Wald Stat = 2.1, *P* = 0.15) or by the interaction between predation and eutrophication (Wald Stat = 0.3, *P* = 0.6) (Fig. 4c). In mesocosms with the highest temperature regime only 2.5% (± SE 1.8%) of the mosquitoes remained in a larval stage at the end of the experiment, whereas the lowest temperature treatment had 30.3% (± SE 10.3%) of the mosquitoes remaining as larvae.

## Discussion

In this study, we used a novel outdoor experimental approach to assess the single and interactive effects of three important ecological drivers (predation, eutrophication, temperature) on mosquito larval development rate, adult emergence and larval survival. Our results were collected under a semi-realistic setting representing realistic variation in food availability for larval mosquitos (eutrophic *vs* oligotrophic) and the presence or absence of invertebrate predation. We found evidence for strong interactive effects between all three drivers: (i) negative effects of predation on adult mosquito emergence depended on the eutrophication conditions; (ii) positive effects of temperature on larval development rates depend on the eutrophication conditions; (iii) adult emergence was affected independently by temperature, eutrophication and its interaction with predation; but (iv) larval survival was only affected by eutrophication and not by temperature or predation.

Our study shows an interaction between predation and eutrophication on the total number of adult mosquitoes emerging as well as on the probability of emergence. There was a strong negative effect of predation on the emergence of adult mosquitoes, but the effect of predation only occurred in eutrophication treatments, thus suggesting that predators are effective in decreasing mosquito emergence in oligotrophic environments but may increase emergence in eutrophic habitats. The reason for this could be that predators consume the smaller prey first [36] and as such, provide an advantage to the larger larvae by decreasing negative density dependent effects such as interference competition. However, based on our results, we cannot exclude the possibility that *A. varia* is a less effective predator under eutrophic conditions, although this does not seem very likely as the species has been naturally observed in all ponds within the measured range of nutrients. Another possibility is that the larvae react to the presence of predation by modifying their behaviour [37], increasing their trophic activity which decreases the development time needed to reach the adult stage [38]. Whether this interaction between eutrophication and predation is similar for other predators and holds across a range of eutrophication regimes warrants further study. Nevertheless, because breeding habitats of *Ae. aegypti* are often oligotrophic [22, 23], it is likely that predators capable of colonizing these, such as backswimmers like *A. varia*, can play an important role in lowering mosquito numbers of those species under such conditions. However, in habitats with high nutrient pollution, our results suggest that the opposite could occur, where predators increase adult emergence.

Previous studies on the dynamics of wild mosquito populations have highlighted the importance of density dependence (e.g. [39, 40]). Food abundance in breeding habitats may be one of the main ecological mechanisms responsible for density dependent effects [41]. In mosquito habitats where food abundance may be limiting larval growth, density dependent effects are likely to play a more important role than in experiments where food is added *ad libitum* (e.g. [42, 43]). Indeed, these results and our previous work on *Cx. pipiens* [12] strongly suggest that the interaction between food abundance and temperature jointly shapes larval development rate, where development rate is fastest at higher temperatures and in eutrophic conditions where food is plentiful. Given that *Ae. aegypti* often breeds in rainwater or tap water fed containers (e.g. [23, 42]) under oligotrophic conditions, the higher temperatures predicted through global temperature forecast models [44] may not necessarily lead to greater *Ae. aegypti* population growth rates in those habitats. Mosquito populations may also be influenced through mechanisms not considered in this study such as the presence of confamiliar species, pesticide pollution, salinity and habitat drying [22]. Similar experiments like the one described in this paper are, therefore, required to investigate the interactive effects of other bottom-up and top-down drivers of both vector and non-vector mosquito species.

## Conclusions

Here we used *Ae. aegypti* to specifically assess the interactive effects of eutrophication and predation on mosquito population dynamics under four different temperature regimes. We performed a controlled outdoor mesocosm experiment in Kruger Park, South Africa which allowed more natural realism than typically represented by laboratory-based studies. Although our experiment had a confined set of drivers and organisms and may thus overlook modulations from biotic and abiotic variables that would otherwise co-occur in natural ecosystems, results obtained in this study confirm that ecological bottom-up and top-down drivers incongruently govern mosquito population dynamics in *Ae. aegypti*. Specifically, depending on temperature, eutrophication can strongly alter mosquito population dynamics and can inversely affect predator–prey interactions. Our results thereby suggest that nutrient pollution can overrule and inverse biological constraints on natural mosquito abundances. This outcome poses great concerns about the consequences of ongoing release of nutrients in the environment for the dynamics of vector-borne disease.

## Additional file

**Additional file 1: Table S1.** Results from the linear models on adult emergence (A) and development rate (B) and results from the binomial models on the probability of emergence of adult females (C) adult males (D) and the probability that larvae remained in the mesocoms at the end of the experiment (E) Significant effects are depicted in bold. **Figure S1.** Randomized treatments in the mesocosm experiment within each of the three blocks. Each of the 48 individual mesocosm is indicated with S followed by a number (1–48). T refers to the temperature regimes where 1 indicates the lowest temperature and 4 refers to highest temperature. E refers to eutrophication (0: oligotrophic, 1: eutrophic) and P refers to predation (0 is absent, 1 is present). **Figure S2.** Phosphorus calibration based on dissolved droppings of Impala (*Aepyceros melampus*): *y *= 0.0093x + 0.6374 and *R*^2^ = 0.9084. Using this formula, we calculated the amount of faeces water necessary to mimic the average observed in natural sites (1.025 mg/l). **Figure S3.** Relationship between temperature and larval survival (**a**, **b**) and adult emergence (**c**, **d**) under eutrophic (**a**, **c**) and oligotrophic conditions (**b**, **d**). Black symbols (± SE) refer to the predator treatments, open symbols refer to treatments without predators. Fits are shown for illustrative purposes only: solid lines indicate significant correlations, dotted lines indicate non-significant correlations. Goodness-of-fit and significance of the fit is given in the upper left corner of each of the panels.
