# Supplementary material for: Eutrophication governs predator-prey interactions and temperature effects in Aedes aegypti populations
Source: Parasit Vectors. 2019 Apr 24;12:179. doi: 10.1186/s13071-019-3431-x (PMC6480876; doi:10.1186/s13071-019-3431-x)
Supplement: Supplementary file 1 — Additional file 1: Table S1. Results from the linear models on adult emergence (A) and development rate (B) and results from the binomial models on the probability of emergence of adult females (C) adult males (D) and the probability that larvae remained in the mesocoms at the end of the experiment (E) Significant effects are depicted in bold. Figure S1. Randomized treatments in the mesocosm experiment within each of the three blocks. Each of the 48 individual mesocosm is indicated with S followed by a number (1–48). T refers to the temperature regimes where 1 indicates the lowest temperature and 4 refers to highest temperature. E refers to eutrophication (0: oligotrophic, 1: eutrophic) and P refers to predation (0 is absent, 1 is present). Figure S2. Phosphorus calibration based on dissolved droppings of Impala (Aepyceros melampus): y = 0.0093x + 0.6374 and R2 = 0.9084. Using this formula, we calculated the amount of faeces water necessary to mimic the average observed in natural sites (1.025 mg/l). Figure S3. Relationship between temperature and larval survival (a, b) and adult emergence (c, d) under eutrophic (a, c) and oligotrophic conditions (b, d). Black symbols (± SE) refer to the predator treatments, open symbols refer to treatments without predators. Fits are shown for illustrative purposes only: solid lines indicate significant correlations, dotted lines indicate non-significant correlations. Goodness-of-fit and significance of the fit is given in the upper left corner of each of the panels. [file 13071_2019_3431_MOESM1_ESM.docx]

**Additional file 1**

*Krol, Gorsich, Hunting, v Bodegom and Schrama.* Eutrophication mediates consequences of predator-prey interactions and temperature for *Aedes aegypti* populations.

*Table S1. Results from the linear models on adult emergence (A) and development rate (B) and results from the binomial models on the probability of emergence of adult females (C) adult males (D) and the probability that larvae remained in the mesocoms at the end of the experiment (E) Significant effects are depicted in bold.*

| A) |  |  |  |  |  |  |  |  |  |
| --- | --- | --- | --- | --- | --- | --- | --- | --- | --- |
| Factor | SS | Degr. of Freedom | MS | F | p | Estimate | SE | Lower CL | Upper CL |
| **Intercept** | **0.13** | **1.00** | **0.13** | **1479.22** | **0.00** | **0.08** | **0.00** | **0.07** | **0.08** |
| Block | 0.00 | 2.00 | 0.00 | 0.27 | 0.77 | 0.00 | 0.00 | 0.00 | 0.01 |
| **Temp** | **0.00** | **3.00** | **0.00** | **5.78** | **0.00** | **-0.01** | **0.00** | **-0.02** | **0.00** |
| Eutrophication | **0.00** | **1.00** | **0.00** | **8.78** | **0.01** | **-0.01** | **0.00** | **-0.01** | **0.00** |
| Predation | 0.00 | 1.00 | 0.00 | 1.55 | 0.23 | 0.00 | 0.00 | -0.01 | 0.00 |
| **Temp*Eut** | **0.00** | **3.00** | **0.00** | **6.09** | **0.00** | **0.02** | **0.00** | **0.01** | **0.03** |
| Pred*Temp | 0.00 | 3.00 | 0.00 | 0.72 | 0.55 | 0.00 | 0.00 | -0.01 | 0.01 |
| Pred*Eut | 0.00 | 1.00 | 0.00 | 1.30 | 0.27 | 0.00 | 0.00 | -0.01 | 0.00 |
| Error | 0.00 | 21.00 | 0.00 |  |  |  |  |  |  |
|  |  |  |  |  |  |  |  |  |  |
|  |  |  |  |  |  |  |  |  |  |
| B) |  |  |  |  |  |  |  |  |  |
| Factor | SS | Degr. of Freedom | MS | F | p | Estimate | SE | Lower CL | Upper CL |
| **Intercept** | **3.72** | **1.00** | **3.72** | **61.16** | **0.00** | **0.28** | **0.04** | **0.21** | **0.35** |
| Block | 0.04 | 2.00 | 0.02 | 0.29 | 0.75 | 0.02 | 0.06 | -0.10 | 0.13 |
| **Temp** | **0.70** | **3.00** | **0.23** | **3.81** | **0.02** | **-0.17** | **0.06** | **-0.30** | **-0.04** |
| Eutrophication | 0.19 | 1.00 | 0.19 | 3.07 | 0.09 | -0.06 | 0.04 | -0.14 | 0.02 |
| Predation | 0.02 | 1.00 | 0.02 | 0.26 | 0.61 | 0.02 | 0.04 | -0.06 | 0.10 |
| Temp*Eut | 0.16 | 3.00 | 0.05 | 0.88 | 0.46 | -0.05 | 0.06 | -0.18 | 0.08 |
| Pred*Temp | 0.17 | 3.00 | 0.06 | 0.91 | 0.45 | -0.06 | 0.06 | -0.18 | 0.07 |
| **Pred*Eut** | **0.37** | **1.00** | **0.37** | **6.02** | **0.02** | **0.08** | **0.04** | **0.01** | **0.16** |
| Error | 2.02 | 33.00 | 0.06 |  |  |  |  |  |  |
|  |  |  |  |  |  |  |  |  |  |
|  |  |  |  |  |  |  |  |  |  |
| C) |  |  |  |  |  |  |  |  |  |
| Factor | Log  likelyhood | Degr. of Freedom | Chi^2^ stat | p | Estimate | Standard Error | Wald  stat | Lower CL | Upper CL |
| **Intercept** | **1.00** | **-32.90** |  |  | **5.09** | **2.51** | **4.11** | **0.17** | **10.01** |
| Block | 2.00 | -31.05 | 3.68 | 0.16 | 0.37 | 0.55 | 0.46 | -0.71 | 1.45 |
| **Temp** | **1.00** | **-25.51** | **4.67** | **0.03** | **-0.22** | **0.10** | **4.65** | **-0.42** | **-0.02** |
| **Eut** | **1.00** | **-27.85** | **4.66** | **0.03** | **0.03** | **2.40** | **0.00** | **-4.67** | **4.73** |
| Pred | 1.00 | -30.17 | 1.76 | 0.19 | 0.52 | 2.34 | 0.05 | -4.07 | 5.11 |
| Temp*Eut | 1.00 | -22.87 | 0.13 | 0.72 | 0.03 | 0.10 | 0.13 | -0.15 | 0.22 |
| Pred*Temp | 1.00 | -22.94 | 0.17 | 0.68 | -0.03 | 0.09 | 0.12 | -0.21 | 0.15 |
| **Pred*Eut** | **1.00** | **-23.02** | **4.98** | **0.03** | **-0.78** | **0.42** | **3.45** | **-1.60** | **0.04** |
|  |  |  |  |  |  |  |  |  |  |
| *Table S1 (continued)*  D) |  |  |  |  |  |  |  |  |  |
| Factor | Log  likelyhood | Degr. of Freedom | Chi^2^ stat | p | Estimate | Standard Error | Wald  stat | Lower CL | Upper CL |
| Intercept | 1.00 | -22.24 |  |  | 0.56 | 3.06 | 0.03 | -5.43 | 6.55 |
| Block | 2.00 | -22.15 | 0.17 | 0.92 | 0.02 | 0.64 | 0.00 | -1.23 | 1.26 |
| Temp | 1.00 | -17.69 | 1.85 | 0.17 | -0.07 | 0.12 | 0.38 | -0.30 | 0.16 |
| **Eut** | **1.00** | **-18.61** | **6.29** | **0.01** | **4.27** | **3.03** | **1.99** | **-1.67** | **10.21** |
| Pred | 1.00 | -21.76 | 0.79 | 0.37 | 0.36 | 2.71 | 0.02 | -4.96 | 5.67 |
| Temp*Eut | 1.00 | -16.95 | 1.16 | 0.28 | -0.13 | 0.12 | 1.17 | -0.35 | 0.10 |
| Pred*Eut | 1.00 | -17.54 | 0.30 | 0.58 | -0.23 | 0.49 | 0.22 | -1.20 | 0.74 |
| Pred*Temp | 1.00 | -17.53 | 0.02 | 0.89 | -0.03 | 0.10 | 0.06 | -0.23 | 0.18 |
|  |  |  |  |  |  |  |  |  |  |
| E) |  |  |  |  |  |  |  |  |  |
| Factor | Log  likelyhood | Degr. of Freedom | Chi^2^ stat | p | Estimate | Standard Error | Wald  stat | Lower CL | Upper CL |
| **Intercept** | **-32.53** | **1.00** |  |  | **-7.69** | **3.24** | **5.62** | **-14.04** | **-1.33** |
| Block | -22.46 | 1.00 | 0.71 | 0.70 | 0.27 | 0.54 | 0.25 | -0.78 | 1.32 |
| **Temp** | **-26.49** | **1.00** | **6.17** | **0.01** | **0.34** | **0.14** | **5.64** | **0.06** | **0.61** |
| **Eut** | **-29.57** | **1.00** | **4.58** | **0.03** | **6.54** | **3.29** | **3.95** | **0.09** | **12.99** |
| Pred | -31.86 | 1.00 | 1.33 | 0.25 | 1.69 | 2.29 | 0.55 | -2.79 | 6.17 |
| **Temp*Eut** | **-22.42** | **2.00** | **7.64** | **0.01** | **-0.31** | **0.14** | **4.69** | **-0.59** | **-0.03** |
| Pred*Eut | -26.34 | 1.00 | 0.31 | 0.58 | 0.25 | 0.47 | 0.29 | -0.66 | 1.17 |
| Pred*Temp | -26.24 | 1.00 | 0.18 | 0.67 | -0.10 | 0.09 | 1.08 | -0.28 | 0.09 |

| Block 1 | | Block 2 | | Block 3 | |
| --- | --- | --- | --- | --- | --- |
| S8  (T4, E2, P1) | S16  (T1, E2, P1) | S24  (T3, E2, P2) | S32  (T4, E1, P1) | S40  (T3, E1, P2) | S48  (T1, E2, P1) |
| S7  (T1, E2, P2) | S15  (T3, E1, P1) | S23  (T2, E2, P2) | S31  (T1, E2, P1) | S39  (T1, E1, P2) | S47  (T1, E1, P1) |
| S6  (T1, E1, P2) | S14  (T2, E1, P2) | S22  (T2, E1, P1) | S30  (T4, E1, P2) | S38  (T4, E1, P2) | S46  (T3, E2, P2) |
| S5  (T4, E1, P2) | S13  (T3, E2, P2) | S21  (T2, E2, P1) | S29  (T3, E1, P1) | S37  (T2, E2, P2) | S45  (T4, E2, P2) |
| S4  (T4, E2, P2) | S12  (T2, E2, P1) | S20  (T1, E1, P2) | S28  (T4, E2, P1) | S36  (T2, E2, P1) | S44  (T2, E1, P1) |
| S3  (T1, E1, P1) | S11  (T2, E1, P1) | S19  (T3, E1, P2) | S27  (T4, E2, P2) | S35  (T4, E1, P1) | S43  (T1, E2, P2) |
| S2  (T3, E2, P1) | S10  (T3, E1, P2) | S18  (T3, E2, P1) | S26  (T2, E1, P2) | S34  (T4, E2, P1) | S42  (T3, E2, P1) |
| S1  (T2, E2, P2) | S9  (T4, E1, P1) | S17  (T1, E1, P1) | S25  (T1, E2, P2) | S33  (T2, E1, P2) | S41  (T3, E1, P1) |

## Figure S1. Randomized treatments in the mesocosm experiment within each of the three blocks. Each of the 48 individual mesocosm is indicated with S followed by a number (1-48). T refers to the temperature regimes where 1 indicates the lowest temperature and 4 refers to highest temperature. E refers to eutrophication (0: oligotrophic, 1: eutrophic) and P refers to predation (0 is absent, 1 is present).

*Median field value*

Figure S2. Phosphorus calibration based on dissolved droppings of Impala (*Aepyceros melampus*): *y* = 0.0093x + 0.6374 and R² = 0.9084. Using this formula we calculated the amount of faeces water necessary to mimic the average observed in natural sites (1.025 mg/l).

$$P concentration (\frac{mg}{l})=0.0093x + 0.6374$$

$$1.025=0.0093x + 0.6374$$

$$x \approx50 ml$$

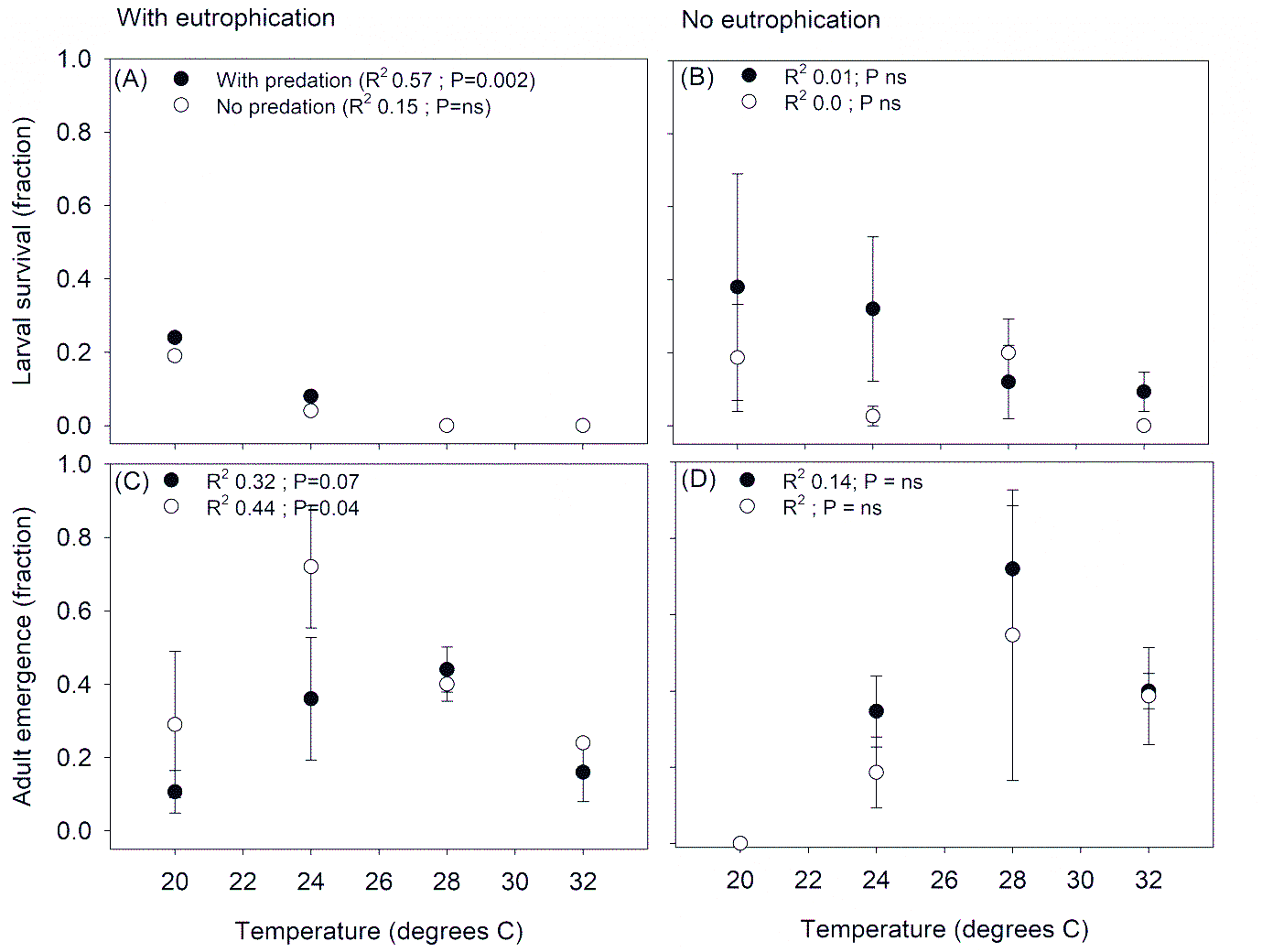


Figure S3. Relationship between temperature and larval survival (A, B) and adult emergence (C.D) under eutrophic (A, C) and oligotrophic conditions (B,D). Black symbols (± SE) refer to the predator treatments, open symbols refer to treatments without predators. Fits are shown for illustrative purposes only: solid lines indicate significant correlations, dotted lines indicate non-significant correlations. Goodness of fit and significance of the fit is given in the upper left corner of each of the panels.
